# Effects of distribution of sugars and organic acids on the taste of strawberries

**DOI:** 10.1002/fsn3.1109

**Published:** 2019-06-13

**Authors:** Atsushi Ikegaya, Tomoyasu Toyoizumi, Seiji Ohba, Teruko Nakajima, Tomoaki Kawata, Seiko Ito, Eiko Arai

**Affiliations:** ^1^ Shizuoka Prefectural Research Institute of Agriculture and Forestry Iwata Japan; ^2^ Graduate School of Integrated Pharmaceutical and Nutritional Sciences University of Shizuoka Shizuoka Japan; ^3^ School of Food and Nutritional Sciences University of Shizuoka Shizuoka Japan

**Keywords:** sensory evaluation, simulated fruit juice jelly, strawberry

## Abstract

The concentrations of sugars and organic acids as well as the total soluble solid (TSS) in different parts of the strawberry fruit were characterized. The data were used to create simulated fruit juice jellies, in order to clarify how the sugar and organic acid levels affect the taste. Such an approach eliminates the influence of external factors such as size, color, and texture when using real fruits in sensory evaluations. Further, the use of a jelly allowed us to simulate the concentration differences between various parts of the fruit. In the strawberry fruit, the sugar content is higher in the apex than in the peduncle; however, the level of organic acids is the same throughout. It was revealed that the sweetness and sourness in the apex and peduncle could be sufficiently recognized by humans as tastes. Also, a layered jelly sample replicating the sugar and acid distribution in real strawberry was perceived as less sweet and more sour, compared to a homogeneous one with the same overall composition. The likely reason is that the sourness in the peduncle is accentuated by the low TSS level, which decreases the TSS/total organic acid ratio that affects the sweetness/sour perceptions. Based on these results, factors for the appropriate sensory evaluation of fresh fruits in general were considered. Specifically, the distribution of sugars and organic acids in the fruit should be analyzed first, and bite‐sized parts with concentrations close to the average provide the most accurate evaluation results.

## 1 INTRODUCTION

Strawberries (*Fragaria x ananassa* Duchesne) were first bred in Dutch farms in the 18th century and have spread worldwide. Various cultivars and methods of cultivation have been developed to adapt them to the environment in each region (Darrow, 1966). To evaluate the various aspects of strawberries, such as improving the cultivars (Ariza et al., 2018), cultivation (Sønsteby, Opstad, Myrheim, & Heide, 2009), preservation (Ayala‐Zavala, Wang, Wang, & González‐Aguilar, 2004; Jiang, Shiina, Nakamura, & Nakahara, 2010), and their processing (Aaby, Grimsbo, Hovda, & Rode, 2018; Kovačević et al., 2015), their “deliciousness” is an extremely important factor.

However, it is very difficult to evaluate the taste of fresh fruits in general, because fruits can vary significantly in their size, color, total soluble solid (TSS), and organic acid contents even when cultivated under the same conditions. Hence, multiple homogeneous samples cannot be obtained, and sensory evaluation has to be performed with nonuniform ones. Moreover, even within a single fruit there are big differences in glucose and sucrose contents among the various parts (Biais et al., 2010). These factors must be adequately considered when performing sensory evaluation on fruits.

Besides the content and composition of sugars and organic acids which constitute taste, the palatability of fruit is affected by many other factors like texture, flavor, color, and size (Banerjee, Tudu, Bandyopadhyay, & Bhattacharyya, 2016; Keast & Breslin, 2002; Yin, Hewson, Linforth, Taylor, & Fisk, 2017). Thus, even though different cultivars, methods of cultivation, and preservation can give rise to differences in palatability, it is difficult to specify the primary cause.

In this study, we aim to clarify the effects of sugars and organic acids, which are the main components found in strawberry fruit juice (Koyuncu & Dilmaçünal, 2010; Paparozzi et al., 2018), on the taste of strawberries. The strawberry fruits were separated into different parts, and the contents of TSS, sugar, and organic acids in each part were analyzed. Next, in order to isolate the taste effects of sugar and organic acid content in different parts of the strawberry, we created simulated fruit juice jellies based on the composition measured in real juice for use in sensory evaluation. Note that by creating a jelly with a coagulant, we were able to replicate the distribution of various components within the fruit.

## 2 MATERIALS AND METHODS

### 2.1 Analysis of TSS, sugar, and organic acid contents in the strawberry fruit

#### 2.1.1 Strawberry fruits

Fresh strawberries (cv. “Benihoppe”) were cultivated in a greenhouse at the Shizuoka Prefectural Research Institute of Agriculture and Forestry. The seedlings were planted in September 2017, and the fruits were harvested in April the following year. The plants were cultivated based on the standard method for Benihoppe (Takeuchi, 2016).

#### 2.1.2 Distribution of TSS within the strawberry fruits

**Figure 1:**
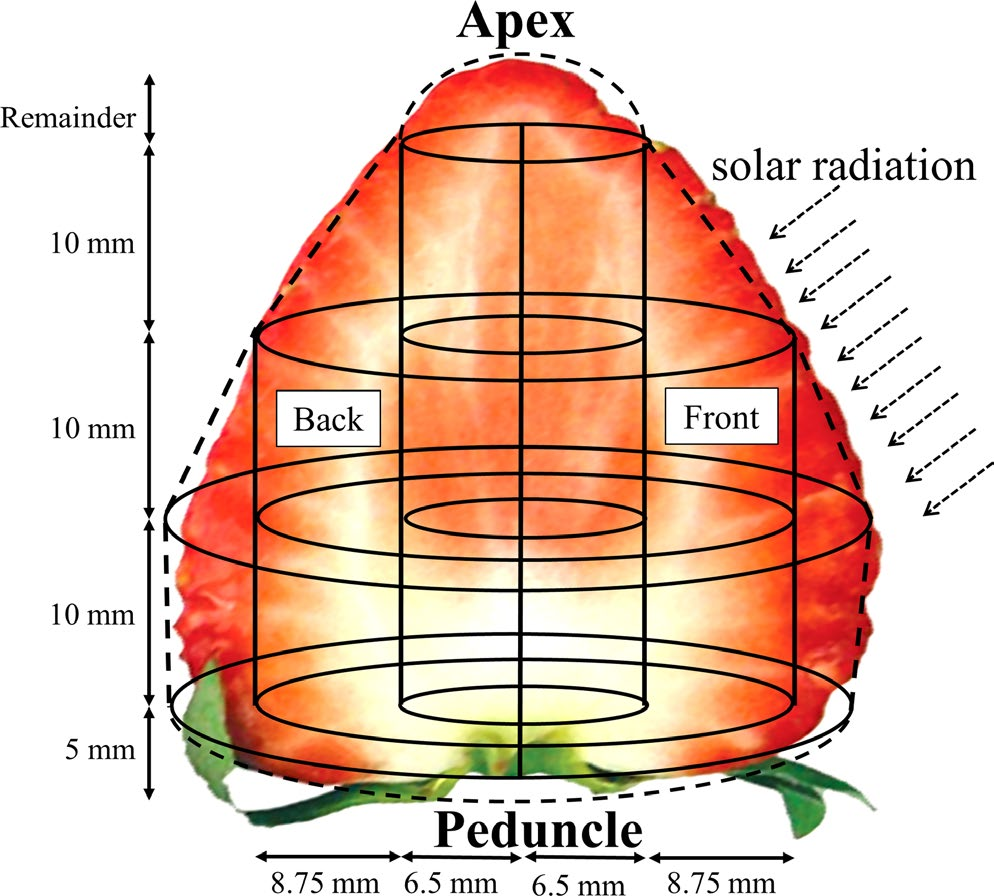
Method of division the strawberry fruit for total soluble solid measurement. “front” is the surface that was predominantly exposed to the sun during cultivation, as opposed to the “back”

Fully ripened strawberries were harvested, and 20 individual fruits weighing 19–23 g each were selected. Figure 1 shows the method of separating each fruit. After removing the hull, the strawberry was sliced into five pieces: 5, 10, 10, and 10 mm in thickness plus the remainder, from the peduncle to the apex. The center of each 10‐mm‐thick piece was punched out using cork borers of diameters 13 and 20.5 mm into annular and cylindrical pieces. Thereafter, each section except the apex was separated into two sides, one received heavy sunlight during cultivation (front) and the other side faced inward (back). In this way, a single strawberry was divided into 19 parts. The segments were wrapped in 100 mesh gauze cloth and squeezed by hand. TSS (Brix) in the resulting juice was measured using a sugar refractometer (SMART‐1, Atago Co., Ltd.) at 20°C.

#### 2.1.3 Sugar and organic acid composition within strawberry fruits

**Figure 2:**
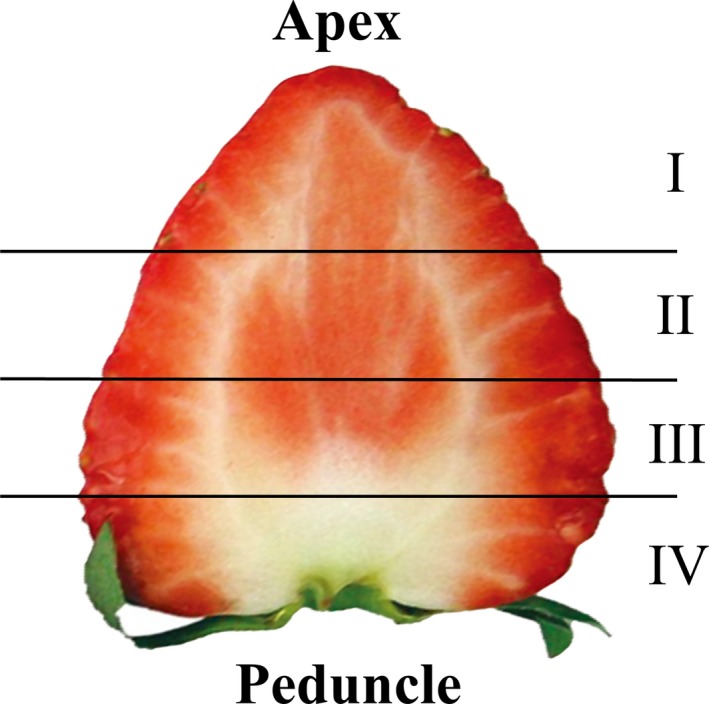
Method of division the strawberry fruit for sugars and organic acids analysis

Each of six fully ripened strawberries was separated from the apex into regions I, II, III, and IV with equal mass (Figure 2). The segments were squeezed and the TSS in the juice was measured, in the same way as in Section 2.1.2. The juice was thereafter subjected to refrigerated centrifugation for 10 min at 6,400 × *g* at 4°C. The supernatant was collected, diluted 50 fold in water, and then filtered through a 0.45 µm filter. The levels of glucose, fructose, and sucrose, which are the main free sugars in strawberries (Paparozzi et al., 2018), were determined in the filtered supernatant using high‐performance liquid chromatography (HPLC). The analysis conditions were as follows: HPLC: Alliance2695 (Waters); column: SC1011 (8 mm I.D. × 300 mm, Showa Denko K.K.); eluent: water; flow rate: 1 ml/min; detector: differential refractometer (2,414, Waters); and standard: glucose, fructose, and sucrose (Wako Pure Chemical Industries).

The same juice supernatant was diluted 5 fold in water, filtered, and used for determining the levels of citric acid and malic acid, which are the main organic acids in strawberries (Koyuncu & Dilmaçünal, 2010), along with succinic acid using HPLC. The analysis conditions were as follows: HPLC: LC‐10AD‐Vp (Shimadzu Corporation); column: Shim‐pack SCR102H (8 mm I.D. × 300 mm, Shimadzu); eluent: 5 mM *p*‐toluenesulfonic acid aqueous solution; flow rate: 0.8 ml/min; detector: conductivity detector (CDD‐6A, Shimadzu); and standard: citric acid, malic acid, and succinic acid (Wako Pure Chemical Industries).

### 2.2 Sensory evaluation using simulated fruit juice jellies

**Figure 3:**
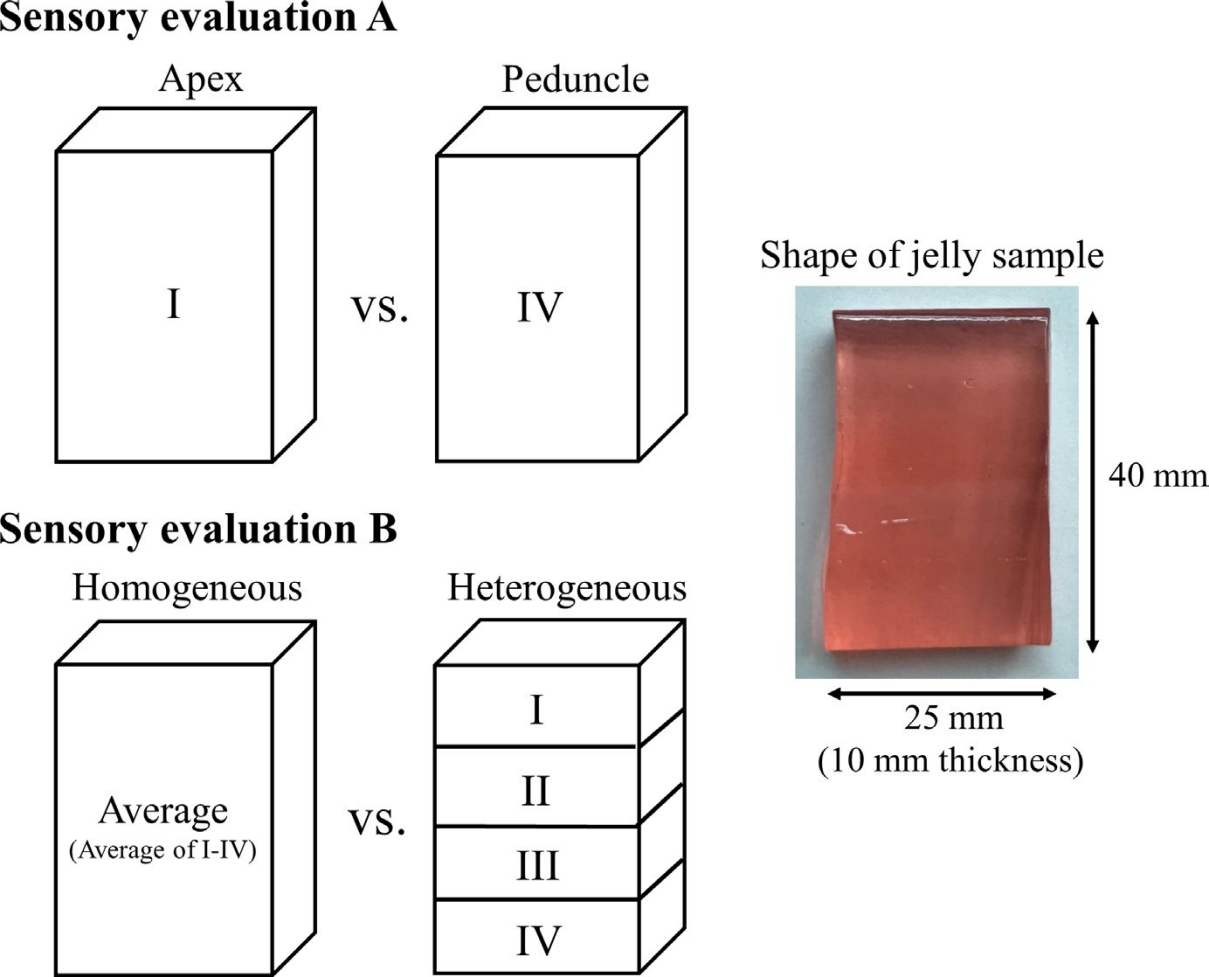
Combinations of the simulated strawberry fruit juice jelly for sensory evaluation

In order to clarify the effects of spatial distribution of sugars and organic acids within the strawberry fruit on its taste, we conducted two sensory evaluations (A and B, Figure 3).

#### 2.2.1 Sensory evaluation using simulated fruit juice jelly replicating *compositions in the peduncle and apex* (sensory evaluation A)

Firstly, in order to verify whether the different concentrations of sugars and organic acids between the apex and peduncle could be recognized in terms of taste, we prepared simulated fruit juice jelly replicating the sugar and organic acid contents in these parts and performed functional evaluation on them.

Table 1 shows the composition of the jellies used in the experiment, where all the ingredients were either foodstuffs or food additives. Glucose (Kanematsu Ensyou Co., Ltd.), fructose (Nissin Sugar Co., Ltd.), sucrose (Mitsui Sugar Co., Ltd.), citric acid (Benisei Co., Ltd.), and malic acid (Happo Shokusan Co., Ltd.), as well as strawberry flavor (Strawberry essence, Golden Kelly Pat. Flavor Co., Ltd.) and red food coloring (Anthocya red RS, Glico Nutrition Co., Ltd.), were dissolved in distilled water to replicate the sugar and organic acid concentrations measured for the fruit apex (I) and the peduncle (IV). A coagulant (Cleagar 100, Aobakasei Co., Ltd.) was added at 3% weight to the mixture and heated to 80°C. Since the coagulant was chiefly carrageenan and roast bean gum, it was both tasteless and odorless.

**Table 1:** Composition of simulated strawberry fruit juices

Ingredient		Concentration (g/L)				
		Part				
		I (Apex)	II	III	IV (Peduncle)	Average^a^
Sugar	Glucose	24.6	21.6	19.0	15.5	20.2
	Fructose	26.4	23.1	20.4	17.0	21.7
	Sucrose	31.8	29.7	26.3	21.6	27.4
Organic acid	Citric	7.4	7.3	7.5	9.1	7.8
	Malic	1.9	1.9	1.8	1.8	1.9
Strawberry flavor		0.2				
Red food coloring		0.2				

a Average sugar and organic acid concentrations of I, II, III, and IV. When producing jelly sample from the simulated strawberry fruit juice, a gelatinizing agent. Corresponding to 3% of the juice mass was added.

The loss of mass due to evaporation during heating was corrected by adding water. Thereafter, 810 ml of the liquid was poured into a stainless steel mold with inner dimensions of 150 × 135 × 47 mm and placed in a refrigerator at 4°C for 18 hr. The prepared jelly was cut to a size of 40 × 25 × 10 mm for sensory evaluation.

The taste of the jelly was evaluated by a panel of 31 participants from various age groups (5 in their 20 s, 6 in 30 s, 8 in 40 s, and 12 in 50 s) and both genders (15 female and 16 male) with various educational backgrounds. The jellies were adjusted to a temperature of 20°C and coded with three random digits. The panelists assessed the two attributes of sweetness and sourness with a two‐sample difference test (Meilgaard, Civille, & Carr, 2007) by choosing the sample they felt as sweeter or sourer. The panelists were instructed to take a single bite of 1 piece of jelly when performing the sensory evaluation.

#### 2.2.2 Sensory evaluation of simulated fruit juice jelly replicating the concentration gradient (sensory evaluation B)

Next, we used simulated fruit juice jelly to verify the effect of the concentration gradient of sugars and organic acids within the strawberry fruit on its taste. A jelly with uniform concentrations of sugars and organic acids (homogeneous) was compared to another jelly with nonuniform concentrations based on the real fruit (heterogeneous). The total amount of sugar and organic acid contained in both jellies were the same. The details and composition of the jellies are described in Figure 3 and Table 1, respectively. First, 4 simulated fruit juices with different sugar and organic acid concentrations (I, II, III, and IV) were prepared using the same method as in Section 2.2.1. After adding the coagulant, the mixture was heated, during which water was added for weight adjustment. Then, the four liquids (202.5 ml each) were poured sequentially after solidification in the order of IV–III–II–I into the same mold, to produce a jelly with layers with different sugar and organic acid concentrations (heterogeneous). A jelly with uniform composition (i.e., the average sugar and organic acid concentrations of I, II, III, and IV) was also produced (homogeneous). The same sample was then divided into four and solidified as a 4‐layer jelly. The prepared jelly was cut in the same way as in Section 2.2.1 into 40 × 25 × 10 mm before sensory evaluation using the same method as in 2.2.1.

### 2.3 Statistical analysis

Each value was expressed as the mean ± standard deviation (*SD*). Results were evaluated using ANOVA, and the means were compared using Tukey’s multiple range test. Paired *t* tests were used to compare TSS in the strawberry’s front and back sides. And the results of sensory evaluation were evaluated by two‐tailed test. All tests used the significance level of 5%.

## 3 RESULTS AND DISCUSSIONS

### 3.1 Total soluble solid distribution within strawberry fruits

**Figure 4:**
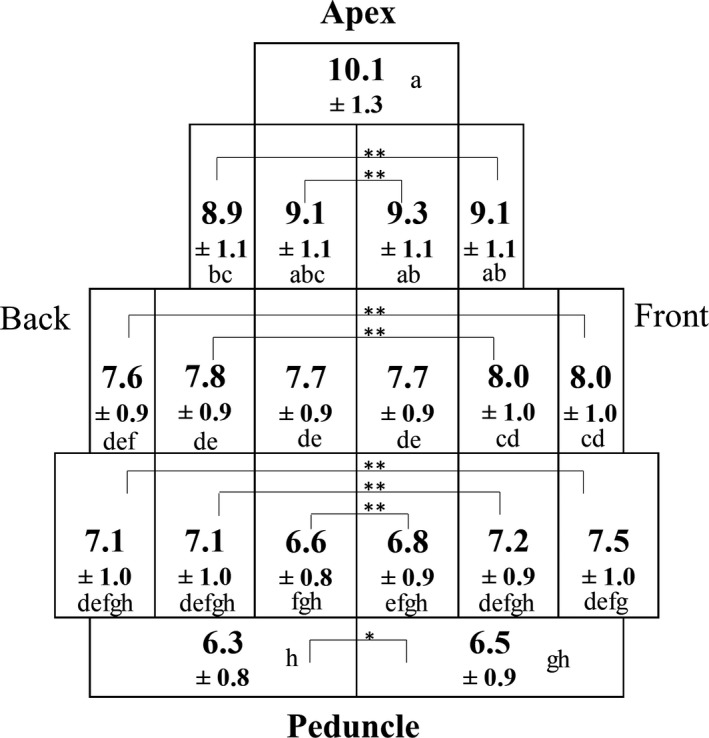
Total soluble solid (Brix) distribution of strawberry fruit. The meaning of “front” and “back” are the same as in Figure 1. The values are presented as mean ± *SD*. (*n* = 20). Mean within a figure followed by different letters are significantly different according to Tukey’s multiple range test at *p* < 0.05. In addition, paired *t* test between “front” and “back” for each part are performed. * and ** are significantly different at *p* < 0.05 and *p* < 0.01, respectively

Figure 4 shows the TSS of each part of the strawberry. The TSS concentration varies greatly between the apex and the peduncle: while the apex’s TSS level was 10.1%, that in the peduncle was lower (6.3% in the back and 6.5% in the front). The sugar content varies greatly within the same strawberry, and this tendency is also reported in melon (Biais et al., 2010).

During cultivation, the surface of strawberry receiving much sunlight becomes darker, and in Japan, this side is called the “front” while the reverse side is called the “back.” The TSS in the front tends to be higher than that in the back. However, this difference is slight with a maximum value of 0.4%, while the difference in TSS between the apex and the peduncle is 3.0%.

### 3.2 Distribution of sugars and organic acids by part of the strawberry

The results in Section 3.1 revealed a large change in TSS within the strawberry fruit from the apex toward the peduncle. Therefore, the mass between the apex and peduncle was separated into four parts equal in weight. The TSS, sugar, and organic acid concentrations of the fruit juice from each part were analyzed, and the results are shown in Table 2.

**Table 2:** TSS, free sugar and organic acid content in parts I‐IV of strawberry fruit, and TSS/TOA rati

Part	TSS (Brix)	Free sugar (g/L)				Organic acid (g/L)			TSS/TOA ratio
		Glucose	Fructose	Sucrose	Total (TS)	Citric	Malic	Total (TOA)	
I	10.0 ± 0.7a	24.6 ± 1.3a	26.4 ± 1.1a	31.8 ± 5.3a	82.8 ± 7.5a	7.4 ± 1.2a	1.9 ± 0.5a	9.3 ± 1.1a	10.9 ± 1.4ab
II	8.6 ± 1.3bc	21.6 ± 0.9b	23.1 ± 1.0b	29.7 ± 4.8ab	74.4 ± 6.0ab	7.3 ± 1.3a	1.9 ± 0.5a	9.1 ± 1.3a	9.5 ± 1.3bc
III	7.8 ± 1.1cd	19.0 ± 1.3c	20.4 ± 1.4c	26.3 ± 4.7ab	65.7 ± 6.2bc	7.5 ± 1.3a	1.8 ± 0.4a	9.3 ± 1.1a	8.5 ± 1.1c
IV	7.0 ± 0.9d	15.5 ± 1.9d	17.0 ± 1.8d	21.6 ± 5.4b	52.2 ± 8.8c	9.0 ± 1.3a	1.8 ± 0.4a	10.9 ± 1.2a	6.5 ± 0.9d

Note

The results are presented as mean ± *SD* (*n* = 6). Values marked with different letters within the same column are significantly different according to Tukey’s multiple range test at *p* < 0.05.

Abbreviations: TOA, total organic acid; TSS, total soluble solid.

As mentioned in Section 3.1, TSS within the strawberry fruit gradually decreases from the apex toward the peduncle. The main sugars found in strawberries are glucose, fructose, and sucrose. These sugars were analyzed, and their sum was taken to be the total sugar (TS). The concentrations of glucose, fructose, and TS decreased from the apex to the peduncle. The content of sucrose varies widely, and significant difference can only be identified between regions I and IV.

Citric acid, malic acid, and succinic acid were analyzed as organic acids. Since the level of succinic acid was below 0.1 g/L in all cases, it was considered to have little effect on taste. Rather, the sum of citric acid and malic acid was taken to be the total organic acid (TOA). There was no variation in the concentrations of citric acid, malic acid, or TOA within the fruit.

The TSS/titratable acidity (TA) ratio can be used to evaluate the sweetness of sour fruits like citrus and strawberries (Fawole & Opara, 2013; Qiu, Wang, & Gao, 2015; Zhou et al., 2018). In this study, since acid was analyzed by HPLC instead of titration, sweetness was evaluated using TOA instead of TA. Because the TSS in strawberries gradually decreases from the apex down to the peduncle (Yoshida, 2016) while the TOA remains constant, the TSS/TOA ratio similarly declines along this direction. The difference in taste from the apex to the peduncle of the strawberry is therefore expected to be larger than the change in sugar content. The fact that sugar and TSS decrease from the apex to the peduncle while the concentration of organic acid remains constant is a new discovery in this research. In addition, the distribution trends of sugars and organic acids are substantially different, suggesting that the metabolic mechanisms relating to their accumulation are also different. We wish to further investigate the root cause of these findings in the future.

### 3.3 Effects of quantity and composition of sugars and organic acids on the taste of strawberry (sensory evaluation A)

After revealing the large difference in the quantity of sugars and TSS/TOA ratio in strawberry fruit, between the apex and the peduncle, next we investigated how this difference affects the taste as experienced by actual people through sensory evaluation. To exclude the effect of individual differences between strawberries, we used model systems and simulated samples for sensory analysis. Evaluation of foodstuffs using model systems had been carried out for wines and cheeses (Arvisenet et al., 2019; Niimi et al., 2016); however, there are no examples pertaining to fresh fruits.

First, we compared the sweetness and sourness of simulated fruit jellies that reproduce the sugar and organic acid compositions in the apex and peduncle of the real fruit. From the results in Table 3, the jelly simulating the apex was considered to have significant strong sweetness and weak sourness. In Section 3.2, the different distribution trends of sugars and organic acids within the strawberry, as well as differences in TSS and the TSS/TOA ratio, have been discussed. The apex of strawberries is generally said to be sweeter and the peduncle more sour (Yoshida, 2016). However, other taste components and sensory elements like flavor and texture may also contribute to the sweetness of strawberries besides sugars and organic acids (Banerjee et al., 2016; Keast & Breslin, 2002; Yin et al., 2017). By using simulated fruit juice jellies, the effects of flavor, texture, and other ingredients within the fruit juice are excluded, and the focus is on whether the different quantity and composition of sugars and organic acids between the apex and the peduncle can be fully perceived as sweetness and/or sourness.

**Table 3:** Results of sensory evaluation A of simulated strawberry fruit juice jellies based on component analysis of fruit apex and peduncle region

Attributes	Apex (I)	Peduncle (IV)	Statistical analysis
Sweetness	27	4	***
Sourness	8	23	*

Note

“Sweetness” and “sourness” are investigated in terms of which one felt stronger. The results are presented as number of responses (*n* = 31).

* and

*** are significantly different according to two‐tailed test at *p* < 0.05 and *p* < 0.001, respectively.

Furthermore, since a strawberry usually cannot be eaten in a single bite, the part that is tasted first will affect the perception of the other parts. For example, after tasting the very sweet apex, taste adaptation will make it hard to evaluate sweetness in the peduncle, and so the acidity will be felt more strongly (Theunissen, Polet, Kroeze, & Schifferstein, 2000). Conversely, if the peduncle is consumed first, the apex can be expected to appear sweeter than when tasted alone.

It was clarified in Sections 3.1, 3.2, 3.3 that there were large differences in the distribution of sugars and organic acids within the strawberry fruit. These differences are expected to affect the taste as in other fruits. Therefore, to exclude the external influences in the sensory evaluation of fresh fruits, it is preferable to first investigate the TSS distribution within the fruit and then cut the fruit into single‐bite pieces with similar TSS concentrations prior to the testing.

### 3.4 Effects of concentration gradient of sugars and organic acids on the taste of strawberry (sensory evaluation B)

Next, two jellies were prepared, one with a uniform concentration (homogeneous) and another containing four layers to replicate the sugar and organic acid distributions as measured in real strawberries (heterogeneous). We compared the strength of sweetness and sourness using sensory evaluation, and Table 4 shows the results. The heterogeneous sample was perceived as having significantly lower sweetness than homogeneous, and a stronger sourness, even though the two jellies had the same total amounts of the various sugars and organic acids. Therefore, the results confirmed that concentration difference in sugars and organic acids has a significant effect on taste.

**Table 4:** Results of sensory evaluation B with different taste homogeneity

Attribute	Homogeneous (Average of I‐IV^a^)	Heterogeneous (4 layers of I‐IV)	Statistical analysis
Sweetness	23	8	*
Sourness	9	22	*

Note

“Sweetness” and “sourness” were investigated in the same as Table 3. The results are presented as number of respondents (*n* = 31).

a As same as Table 1.

* is significantly different according to two‐tailed test at *p* < 0.05.

In the case of saltiness, the taste of substances is known to be felt more strongly when there are differences in their concentration while the total amount is kept the same (Dijksterhuis, Boucon, & Berre, 2014). In the present study for sweetness and sourness, however, heterogeneous was perceived as less sweet and with stronger sourness in sensory evaluation compared with homogeneous. A likely explanation is that, when organic acid is mixed with sugar, the sourness is weakened (Green, Lim, Osterhoff, Blacher, & Nachtigal, 2010). However, since parts III and IV in heterogeneous had little sugar, their sourness was felt more strongly. This result suggests that a stronger sourness through decreased TSS/TA ratio exerts a bigger effect on the strawberry’s taste than stronger sweetness from a higher sugar content. In addition, since the perception threshold for sourness is generally lower than that for sweetness (Amerine, Pangborn, & Roessler, 1965; Pfaffmann, 1959; Yamaguchi, 1979), the sourness effect on the taste of fruit is strong. For the taste of fruits, it is considered important to evaluate it in combination with the TSS/TOA ratio and not by TSS alone.

In Section 3.3, we mentioned that it is desirable to use bite‐sized pieces for sensory evaluation of fresh fruits. Meanwhile, it is even more desirable to perform the test using parts with less variance in sugars and organic acids. Taking the strawberry as an example, even when the fruits can be eaten in a single bite, more accurate sensory evaluation results can be obtained by first removing the parts where the TSS concentration greatly diverges from the average, such as the apex and the peduncle.

## 4 CONCLUSIONS

It is widely known that TSS is higher in the apex of strawberry fruit and lower in the peduncle. In this study, we further discovered that while the sugar content is higher in the apex, the distribution of organic acids in the fruit is almost uniform, and thus, the TSS/TOA ratio varies greatly between parts of the fruit.

To exclude unwanted influencing factors, sensory evaluation were conducted using simulated fruit juice jellies prepared using foodstuffs and food additives, based on the sugar and acid contents measured in real strawberry fruits. The differences in sugar and acid concentrations between the apex and peduncle were found to have recognizable effects on the taste. In the peduncle, the TSS is low and the TSS/TOA ratio is also low, and so the sourness is felt more strongly. Compared with a homogeneous sugar and acid distribution, their concentration gradients throughout the fruit will accentuate the sourness.

This system of evaluation is highly effective for comparing specific elements in fresh fruits by excluding the effects of variations in quality. Since the distribution of sugars and organic acids is not uniform and this affects the taste, it is preferable to first analyze their contents in fresh fruit and then cut the fruit into bite‐sized pieces and use those with sugar and acid concentrations close to the average values for sensory evaluation. Even with fruits that can be eaten in one bite, it is preferable to exclude parts where the TSS greatly diverges from the average value.

In future work, we wish to clarify the effects of flavor, color, and physical properties of strawberries using this evaluation system and carry out more detailed investigation of the effects of sugars and organic acids on taste. We are further interested in developing more appropriate methods to evaluate various fruits besides strawberries.

## CONFLICT OF INTEREST

The authors declare no conflict of interest or relationship, financial, or otherwise.

## ETHICAL APRROVAL

Ethical Review: This study was approved by the Institutional Review Board of Shizuoka Prefectural Research Institute of Agriculture and Forestry.

## INFORMED CONSENT

Informed consent was obtained from all individual participants included in the study.

## References

[fsn31109-bib-0001] Aaby, K. , Grimsbo, I. H. , Hovda, M. B. , & Rode, T. M. (2018). Effect of high pressure and thermal processing on shelf life and quality of strawberry purée and juice. Food Chemistry, 260, 115–123. 10.1016/j.foodchem.2018.03.100 29699651

[fsn31109-bib-0002] Amerine, M. A. , Pangborn, R. M. , & Roessler, E. B. (1965). Principles of sensory evaluation of food In: Stumbo C. R. (Ed.), Food Science and Technology Monographs (pp. 338–339). New York, NY: Academic Press.

[fsn31109-bib-0003] Ariza, M. T. , Reboredo‐Rodríguez, P. , Cervantes, L. , Soria, C. , Martínez‐Ferri, E. , González‐Barreiro, C. , … Simal‐Gándara, J. (2018). Bioaccessibility and potential bioavailability of phenolic compounds from achenes as a new target for strawberry breeding programs. Food Chemistry, 248, 155–165. 10.1016/j.foodchem.2017.11.105 29329839

[fsn31109-bib-0004] Arvisenet, G. , Ballester, J. , Ayed, C. , Sémon, E. , Andriot, I. , Quere, J. L. , & Guichard, E. (2019). Effect of sugar and acid composition, aroma release, and assessment conditions on aroma enhancement by taste in model wines. Food Quality and Preference, 71, 172–180. 10.1016/j.foodqual.2018.07.001

[fsn31109-bib-0005] Ayala‐Zavala, J. F. , Wang, S. Y. , Wang, C. Y. , & González‐Aguilar, G. A. (2004). Effect of storage temperatures on antioxidant capacity and aroma compounds in strawberry fruit. LWT‐Food Science and Technology, 37, 687–695. 10.1016/j.lwt.2004.03.002

[fsn31109-bib-0006] Banerjee, R. , Tudu, B. , Bandyopadhyay, R. , & Bhattacharyya, N. (2016). A review on combined odor and taste sensor systems. Journal of Food Engineering, 190, 10–21. 10.1016/j.jfoodeng.2016.06.001

[fsn31109-bib-0007] Biais, B. , Beauvoit, B. , Allwood, W. , Deborde, C. , Maucourt, M. , Goodacre, R. , … Moing, A. (2010). Metabolic acclimation to hypoxia revealed by metabolite gradients in melon fruit. Journal of Plant Physiology, 167, 242–245. 10.1016/j.jplph.2009.08.010 19781810

[fsn31109-bib-0008] Darrow, G. (1966). The strawberry: History, breeding and physiology. New York, NY: Holt, Rinehart and Winston.

[fsn31109-bib-0009] Dijksterhuis, G. , Boucon, C. , & Berre, E. L. (2014). Increasing saltiness perception through perceptual constancy created by expectation. Food Quality and Preference, 34, 24–28. 10.1016/j.foodqual.2013.12.003

[fsn31109-bib-0010] Fawole, O. A. , & Opara, U. L. (2013). Harvest discrimination of pomegranate fruit: Postharvest quality changes and relationships between instrumental and sensory attributes during shelf life. Journal of Food Science, 72, 1264–1272. 10.1111/1750-3841.12176 23815086

[fsn31109-bib-0011] Green, B. G. , Lim, J. , Osterhoff, F. , Blacher, K. , & Nachtigal, D. (2010). Taste mixture interactions: Suppression, additivity, and the predominance of sweetness. Physiology & Behavior, 101, 731–737. 10.1016/j.physbeh.2010.08.013 20800076 PMC2975745

[fsn31109-bib-0012] Jiang, Y. , Shiina, T. , Nakamura, N. , & Nakahara, A. (2010). Electrical conductivity evaluation of postharvest strawberry damage. Journal of Food Science, 66, 1392–1395. 10.1111/j.1365-2621.2001.tb15220.x

[fsn31109-bib-0013] Keast, R. , & Breslin, P. (2002). An overview of binary taste–taste interactions. Food Quality and Preference, 14, 111–124. 10.1016/S0950-3293(02)00110-6

[fsn31109-bib-0014] Kovačević, D. B. , Putnik, P. , Dragović‐Uzelac, V. , Vahčića, N. , Babojelić, M. S. , & Levaj, B. (2015). Influences of organically and conventionally grown strawberry cultivars on anthocyanins content and color in purees and low‐sugar jams. Food Chemistry, 181, 94–100. 10.1016/j.foodchem.2015.02.063 25794726

[fsn31109-bib-0015] Koyuncu, M. A. , & Dilmaçünal, T. (2010). Determination of vitamin C and organic acid changes in strawberry by HPLC during cold storage. Notulae Botanicae Horti Agrobotanici Cluj‐Napoca, 38, 95–98.

[fsn31109-bib-0016] Meilgaard, M. , Civille, G. , & Carr, T. (Eds.), (2007). Sensory evaluation technique, (4th edn). New York, NY: CRC Press.

[fsn31109-bib-0017] Niimi, J. , Eddy, A. I. , Overington, A. R. , Heenan, S. , Silcock, P. , Bremer, P. J. , & Delahunty, C. M. (2016). Aroma–taste interactions between a model cheese aroma and five basic tastes in solution. Food Quality and Preference, 31, 2419–9. 10.1016/j.foodqual.2013.05.017

[fsn31109-bib-0018] Paparozzi, E. T. , Meyer, G. E. , Schlegel, V. , Blankenship, E. E. , Adams, S. A. , Conley, M. E. , … Reade, P. E. (2018). Strawberry cultivars vary in productivity, sugars and phytonutrient content when grown in a greenhouse during the winter. Scientia Horticulturae, 227, 2419–9. 10.1016/j.scienta.2017.07.048

[fsn31109-bib-0019] Pfaffmann, C. (1959). The afferent code for sensory quality. American Psychologist, 14, 226–232. 10.1037/h0049324

[fsn31109-bib-0020] Qiu, S. , Wang, J. , & Gao, L. (2015). Qualification and quantisation of processed strawberry juice based on electronic nose and tongue. LWT‐Food Science and Technology, 60, 115–123. 10.1016/j.lwt.2014.08.041

[fsn31109-bib-0021] Sønsteby, A. , Opstad, N. , Myrheim, U. , & Heide, O. M. (2009). Application of vermicompost improves strawberry growth and quality through increased photosynthesis rate, free radical scavenging and soil enzymatic activity. Scientia Horticulturae, 204, 204–209.

[fsn31109-bib-0022] Takeuchi, T. (2016). Characteristics of major varieties and cultivation methods “Beni Hoppe” In Strawberry dictionary (pp 367–371). Tokyo, Japan: Rural Culture Association Japan. (In Japanese)

[fsn31109-bib-0023] Theunissen, M. J. M. , Polet, I. A. , Kroeze, J. H. A. , & Schifferstein, H. N. J. (2000). Taste adaptation during the eating of sweetened yogurt. Appetite, 34, 21–27. 10.1006/appe.1999.0275 10744888

[fsn31109-bib-0024] Yamaguchi, S. (1979). The umami taste In Boudreau J. C. (Eds.), Food taste chemistry (pp. 35–51). Washington, D.C.: American Chemical Society.

[fsn31109-bib-0025] Yin, W. , Hewson, L. , Linforth, R. , Taylor, M. , & Fisk, I. D. (2017). Effects of aroma and taste, independently or in combination, on appetite sensation and subsequent food intake. Appetite, 114, 265–274. 10.1016/j.appet.2017.04.005 28396048 PMC5434034

[fsn31109-bib-0026] Yoshida, Y. (2016). Development and quality of fruit . In Strawberry dictionary (pp 89–99). Tokyo, Japan: Rural Culture Association Japan. (In Japanese).

[fsn31109-bib-0027] Zhou, Y. , He, W. , Zheng, W. , Tan, Q. , Xie, Z. , Zheng, C. , & Hu, C. (2018). Fruit sugar and organic acid were significantly related to fruit Mg of six citrus cultivars. Food Chemistry, 259, 278–285. 10.1016/j.foodchem.2018.03.102 29680055

